# Integration of conventional and advanced molecular tools to track footprints of heterosis in cotton

**DOI:** 10.1186/s12864-018-5129-4

**Published:** 2018-10-29

**Authors:** Zareen Sarfraz, Muhammad Shahid Iqbal, Zhaoe Pan, Yinhua Jia, Shoupu He, Qinglian Wang, Hongde Qin, Jinhai Liu, Hui Liu, Jun Yang, Zhiying Ma, Dongyong Xu, Jinlong Yang, Jinbiao Zhang, Wenfang Gong, Xiaoli Geng, Zhikun Li, Zhongmin Cai, Xuelin Zhang, Xin Zhang, Aifen Huang, Xianda Yi, Guanyin Zhou, Lin Li, Haiyong Zhu, Yujie Qu, Baoyin Pang, Liru Wang, Muhammad Sajid Iqbal, Muhammad Jamshed, Junling Sun, Xiongming Du

**Affiliations:** 1 State Key Laboratory of Cotton Biology/Institute of Cotton Research, Chinese Academy of Agricultural Sciences (ICR, CAAS), P. O. Box 455000, Anyang, Henan China; 2 Henan Institute of Science and Technology, Xinxiang, China; 3 Cash Crop Institute, Hubei Academy of Agricultural Sciences, Wuhan, China; 4 Zhongmian Cotton Seed Industry Technology CO., LTD, Zhengzhou, China; 5 Jing Hua Seed Industry Technologies Inc, Jingzhou, China; 6 Cotton Research Institute of Jiangxi Province, Jiujiang, China; 7 Key Laboratory of Crop Germplasm Resources of Hebei, Agricultural University of Hebei, Baoding, China; 8 Guoxin Rural Technical Service Association, Hebei, China; 9 Zhongli Company of Shandong, Shandong, China; 10 Hunan Cotton Research Institute, Changde, China; 11 Sanyi Seed Industry of Changde in Hunan Inc, Changde, China; 12 Cotton Research Station, Ayub Agricultural Research Institute, Faisalabad, Pakistan

**Keywords:** Heterosis, L × T, GCA, SCA, Microsatellite markers, hQTL, Favorable alleles, Fiber quality, Cotton

## Abstract

**Background:**

Heterosis, a multigenic complex trait extrapolated as sum total of many phenotypic features, is widely utilized phenomenon in agricultural crops for about a century. It is mainly focused on establishing vigorous cultivars with the fact that its deployment in crops necessitates the perspective of genomic impressions on prior selection for metric traits. In spite of extensive investigations, the actual mysterious genetic basis of heterosis is yet to unravel. Contemporary crop breeding is aimed at enhanced crop production overcoming former achievements. Leading cotton improvement programs remained handicapped to attain significant accomplishments.

**Results:**

In mentioned context, a comprehensive project was designed involving a large collection of cotton accessions including 284 lines, 5 testers along with their respective F_1_ hybrids derived from Line × Tester mating design were evaluated under 10 diverse environments. Heterosis, GCA and SCA were estimated from morphological and fiber quality traits by L × T analysis. For the exploration of elite marker alleles related to heterosis and to provide the material carrying such multiple alleles the mentioned three dependent variables along with trait phenotype values were executed for association study aided by microsatellites in mixed linear model based on population structure and linkage disequilibrium analysis. Highly significant 46 microsatellites were discovered in association with the fiber and yield related traits under study. It was observed that two-thirds of the highly significant associated microsatellites related to fiber quality were distributed on D sub-genome, including some with pleiotropic effect. Newly discovered 32 hQTLs related to fiber quality traits are one of prominent findings from current study. A set of 96 exclusively favorable alleles were discovered and C tester (A971Bt) posited a major contributor of these alleles primarily associated with fiber quality.

**Conclusions:**

Hence, to uncover hidden facts lying within heterosis phenomenon, discovery of additional hQTLs is required to improve fibre quality. To grab prominent improvement in influenced fiber quality and yield traits, we suggest the A971 Bt cotton cultivar as fundamental element in advance breeding programs as a parent of choice.

**Electronic supplementary material:**

The online version of this article (10.1186/s12864-018-5129-4) contains supplementary material, which is available to authorized users.

## Background

Cotton is a significant agricultural crop with high economic importance acting as a vital source for provision of income to large number of farmers around the world. Presence of diversity as well as agro climatic zones regarding cotton in China are comparably larger than any other country around the globe. Genus *Gossypium* covers economically sustainable and diverse amount of diploid as well as tetraploid cotton species grown in most of the regions worldwide [1]. Approximately 95% of cotton production in the whole world accredited with tetraploid *Gossypium hirsutum* species mostly renowned as ‘upland cotton’. Most of the times breeders concerned with plants face the difficulty in selecting suitable parents and crosses while studying qualitative and quantitative traits responsible for yield.

Based on phenotype only, parent selection procedure may prove faulty as phenotypically superior plants may lead to poor combinations. Integration of knowledge related to genetic basis of yield and quality traits of parents would definitely aid in the identification of superior cross combinations in earlier generations. Although cotton production has flourished significantly in recent decade however, the hybrid cotton yield is now at stagnation phase. Main reasons behind this scenario include lack of organized efforts for developing hybrid populations and derived lines with better combining abilities for establishing subsequent new hybrids.

One of the major breakthrough in crop breeding era is large production of high yielding hybrids through wide exploitation of heterosis. Maize, sunflower, pearl millet, sugarbeet, sorghum and many other vegetables beneficially grown from their respective hybrids. However, areas under cultivation of rice, cotton, rapeseed and safflower are rapidly increasing. In open-pollinated crops such as maize, it is fundamental to establish heterotic populations and set grounds for improvement of combining ability to achieve sustainable productivity [2]. After its initial introduction and description, many researchers worked out intraspecific and interspecific heterosis in cotton [3] regarding fibre quality, reproductive cum vegetative growth and photosynthates manufacturing [4]. Since longer times, producers and researchers are focusing on heterosis to use it as a major tool for raising fibre yield and quality of cotton [5]. Earlier in 1894, heterosis in cotton accounting certain measurements of agronomic and fiber properties, was discovered and reported by Mell [6], then Shull in 1908 [7] gave its modern concept [6]. Hybrids between Upland and Egyptian cotton produce lint of superior quality. As in maize the yield increments are highly correlated with hybrid breeding, a parallel scenario has been observed in cotton. However, for the durable implementation of efficient procedures and basic genetic grounds of hybridization in cotton, much exploration is yet required to fill that gap which is one of the reasons for lagging behind of maize.

China as well as India, both are large consumers of hybrid cotton, which has become possible due to advanced studies on heterosis aspect [8]. Adoption of hybrid cotton is rapidly increasing in China due to commercial release of Bt-cotton varieties. Nowadays hybrids (F_1_) of cotton in China are produced preferably from crossing of a non-Bt cotton line with a Bt cotton line [9]. It has been scientifically proved that such type of crossing gives significant better-parent heterosis or Mid-parent heterosis especially in fiber yield components [10].

By exploiting the ambiguous mechanism of heterosis, many scientists have utilized inbred lines with suitable partners to produce elite hybrids with increased yield in different breeding programs [11]. Therefore, plant breeders examine inbred lines by reviewing their potential to produce elite hybrids and not by their performance per se.

Hybrid performance cannot be precisely analyzed by line performance [12], verifying phenotypic trait assessment of hybrid crosses as liable. Such types of hindrances are typically sorted out by hybridizing inbred lines and ‘testers’ (genetically distant) along with evaluating their (inbred lines) general combining abilities (GCA). Novel implements are required for precise prediction of GCA connected with highly polygenic parameters based on information derived specifically from parental inbred lines [13]. Mating designs play vital role in breeding of crops as they find their deliberate use in estimating GCA and SCA of parents and F_1_s. The line × tester is a simplest and efficient method utilized to breed all types of crop plants either self or cross pollinated in order to evaluate superior parents and favorable crosses along with their GCA and SCA [14]. Many breeding programs utilized this method to achieve hybrid vigor for its commercial use. Analyzing combining ability is essential for the sake of selecting appropriate parents along with facts related to nature as well as extent of gene effects governing polygenic parameters. A successful hybridization program is highly dependent on the capability of parents involved to produce desirable recombinants [2].

Earlier reports unravel that additive and dominance effects laid the foundation of genetics related to heterosis for cotton yield [15, 16]. In previous times, the trait value worked out using classical quantitative genetic methods. Consequently, dominance [17, 18], over-dominance [7, 19] and epistasis [20, 21] hypotheses relating heterosis came into being. With the advent of molecular markers in collaboration with extensive exploitation of QTL mapping for dominance [22], over-dominance [23] as well as epistasis [22] theories greatly reinforced to analyze trait phenotype and heterosis [24].

Plant breeders are working hardly to mine the secrets lying behind this ambiguous process of heterosis, which is truly speaking genetically unclear so far. Many investigations have been conducted so far to explore the genetic grounds of heterosis [25]. Even then, investigators are enjoying the benefits of hybrids by exploiting it. Construction of saturated genetic linkage maps by utilizing molecular markers for the dissection of genetic components responsible for yield related complex traits through QTL (quantitative trait loci) analysis may substantially lead to comprehend the complex process of heterosis. Through association analyses, various yield related aspects of cotton have been mined thoroughly for the identification of significant alleles and carriers for breeding materials [26, 27]. Variations existing in cotton genotypes identified via DNA markers have been related to significant heterosis results in order to utilize them in further hybrid breeding programs [28]. Many researchers in intra as well as inter-specific cotton hybrids for the sake of discovering the affiliation concerning hybrid performance and parental molecular marker diversity [29] have investigated prediction of hybrid performance with the help of molecular markers.

Cotton improvement programs remained handicap to attain significant achievements. We paced in this field for the exploration of elite marker alleles related to heterosis and to provide the typical material carrying such multiple alleles by integrating Line × Tester mating design with microsatellites based genome wide association mapping. Specific objectives of the current project were to investigate population structure of parents and hybrids, to discover the loci in F_1_ hybrid individuals, associated with high heterosis influencing improved fiber yield and to identify elite alleles and the respective materials for further cotton improvement programs aimed at fiber quality and yield.

## Results

### Phenotypic evaluation and population structure

**Fig. 1:**
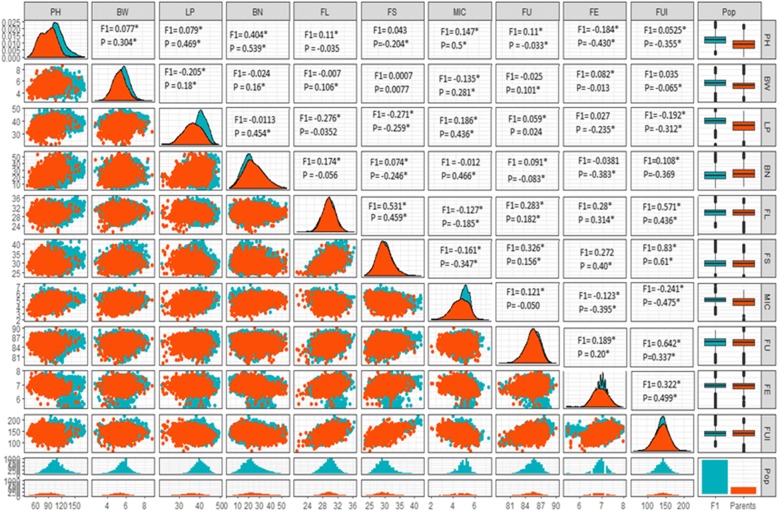
Correlogram for fiber quality traits in F_1_s and Parents of upland cotton. The density distribution of each variable for F_1_s and Parents is shown at diagonal with distinct colors (blue: F_1_s, orange: Parents). On the lower side, the bivariate scatter plots are displayed while on the upper side, the values along with significance (*) of correlation coefficients for variables of F1 s and Parents are presented. Boxplots illustrating the variability among individuals of parents and offsprings. The central box represents the middle half data lengthening from upper to lower quartile while the horizontal line is located at median. The ends point of vertical projections specify maximum and minimum data points, unless the presence of outliers. Solid dots at upper and lower sides represents outliers. The bottom most rows depicted frequency distribution of each variable for F_1_s and Parents

Means and ranges of 10 traits evaluated in the field trials are given in Table 1. All traits showed considerable range of variation among hybrids as well as parental genotypes analyzed. As shown in correlogram the correlation (r) between different agronomic and fibre quality traits of investigated material revealed that plant height displayed positive correlation with BW. BW showed highly significant positive correlation with FUI, FE, FU, MIC, FS, FL, and LP. LP displayed positive correlation with all traits. BN depicted negative correlation with FL. FL showed positive correlation with FS and FUI but negative correlation with MIC. FS exhibited affirmative correlation with FE and FUI whereas negatively correlated with MIC. FUI is positively correlated with FU and FE characteristics while negatively correlated with MIC. Boxplot for all traits are depicting significant variation among individuals of F_1_s and parents (Fig. 1). The central box represents the middle half data lengthening from upper to lower quartile while the horizontal line is located at median. The ends point of vertical projections specifies maximum and minimum data points, unless the presence of outliers. Solid dots at upper and lower sides represents outliers.

**Table 1:** Summary of F_1_ hybrids and parents for phenology and fibre related traits from 2 locations and 2 years

Trait	Loc.	Year	Min.	1st Q	Med.	3rd Q	Max.	Mean	S.D
PH	1	1	42.60	65.60	73.70	81.70	123.70	74.14	11.74
PH	1	2	60.00	93.00	99.50	106.50	139.00	99.90	10.85
PH	2	1	40.30	72.60	79.40	84.85	118.60	78.99	9.62
PH	2	2	88.20	119.00	125.40	134.00	160.00	126.16	11.56
BW	1	1	2.90	4.50	4.90	5.40	7.90	4.99	0.71
BW	1	2	2.70	4.90	5.40	5.70	8.60	5.37	0.71
BW	2	1	3.60	5.00	5.30	5.70	6.90	5.32	0.53
BW	2	2	2.30	4.50	4.90	5.30	7.10	4.90	0.66
LP	1	1	22.30	31.50	34.00	36.20	45.20	33.86	3.44
LP	1	2	22.20	35.80	38.40	40.60	47.40	37.93	3.74
LP	2	1	25.80	33.30	35.30	37.10	44.00	35.14	2.81
LP	2	2	24.70	34.20	36.10	38.00	44.10	36.03	2.81
BN	1	1	3.30	16.20	20.00	23.60	40.70	19.96	5.59
BN	1	2	9.30	26.50	31.10	36.13	57.80	31.69	7.47
BN	2	1	1.60	10.80	14.30	18.30	29.10	14.55	5.23
BN	2	2	0.40	5.60	8.60	12.20	38.00	9.23	4.91
FL	1	1	22.00	28.70	29.90	31.10	85.20	29.91	2.64
FL	1	2	21.50	28.60	29.50	30.60	34.10	29.56	1.59
FL	2	1	21.70	28.90	29.75	30.70	36.30	29.77	1.50
FL	2	2	23.20	29.40	30.50	31.50	37.10	30.44	1.58
FS	1	1	24.10	29.00	30.40	32.00	39.40	30.61	2.28
FS	1	2	23.30	27.40	28.80	30.60	39.30	29.13	2.49
FS	2	1	22.00	26.20	27.40	29.20	37.20	27.82	2.34
FS	2	2	22.20	28.50	30.30	32.20	45.70	30.58	2.98
MIC	1	1	2.10	3.80	4.20	4.60	5.60	4.18	0.56
MIC	1	2	2.60	4.50	5.10	5.50	6.40	4.96	0.66
MIC	2	1	3.20	4.70	5.00	5.30	6.30	4.97	0.47
MIC	2	2	3.20	5.10	5.40	5.60	7.10	5.27	0.54
FU	1	1	79.90	84.60	85.60	86.40	88.50	85.43	1.40
FU	1	2	76.10	84.30	85.30	86.13	89.80	85.14	1.50
FU	2	1	79.30	84.90	85.70	86.60	89.50	85.64	1.34
FU	2	2	80.80	85.60	86.40	87.20	90.80	86.41	1.32
FE	1	1	5.60	6.90	7.10	7.30	7.90	7.10	0.26
FE	1	2	5.60	6.50	6.70	6.80	7.90	6.69	0.25
FE	2	1	5.60	6.50	6.80	7.00	7.90	6.74	0.36
FE	2	2	5.40	6.70	6.80	7.00	7.80	6.81	0.25
FUI	1	1	87.00	142.00	151.00	161.00	205.00	150.97	14.71
FUI	1	2	65.00	119.00	131.00	143.00	189.00	131.38	17.71
FUI	2	1	72.00	127.75	136.00	147.00	193.00	136.77	15.63
FUI	2	2	72.00	146.00	155.00	165.00	226.00	155.93	15.78

Note: 1st Q–25%-ile; 3rd Q–75%-ile

Countless amount of studies from different fields in search of illuminating most of the total phenotypic variance explained by correlated phenotypes; follow the principle of dimension reduction. In order to visualize and verify the connection and variability between phenology of parents and their respective F_1_ hybrids Principal Components Analysis (PCA) performed. It was carried out based on correlation between agronomic and fiber traits. Ten principal components were extracted from the ten studied traits through PCA. The first three principal components were detected to reveal eigen value exceeding 1 while rest of the seven components showed less than one eigen value. The first and second principal components accounted collectively for 18.05% of total variation. The cumulative percent of variance accounted 57.76% of total variation in the first two components (Additional file 1).

Contribution of a specific trait towards variability among PCs unravel that FUI stood first in donating maximum positive loading vector i.e., 0.8921 followed by FS (0.7526), FL (0.6376), MIC (0.5197), LP (0.3752) and BN (0.3515) for first PC. It is described that the mentioned six original variables are strongly correlated with first principal component. It will increase with upgradation in scores of these variables, which suggested that these six criteria vary altogether. FUI was found strongly correlated with this principal component. Indeed, it could be stated that this PC is predominantly a measure of FUI. However, remaining four traits contributed minimum positive loadings.

Net variation displayed by second PC was 18.0540 and maximum loading factor in this PC was exhibited by PH (0.8018) followed by BW (0.7773). Hence, this PC will increase by increase in PH and BW variables as being highly correlated. While remaining eight traits FL, FU, FE, MIC, FS, LP, FUI and BN revealed minimum loadings as 0.0548, 0.0427, 0.0394, 0.0379, 0.0287, 0.0219, 0.0006 and 0.0003 respectively.

**Fig. 2:**
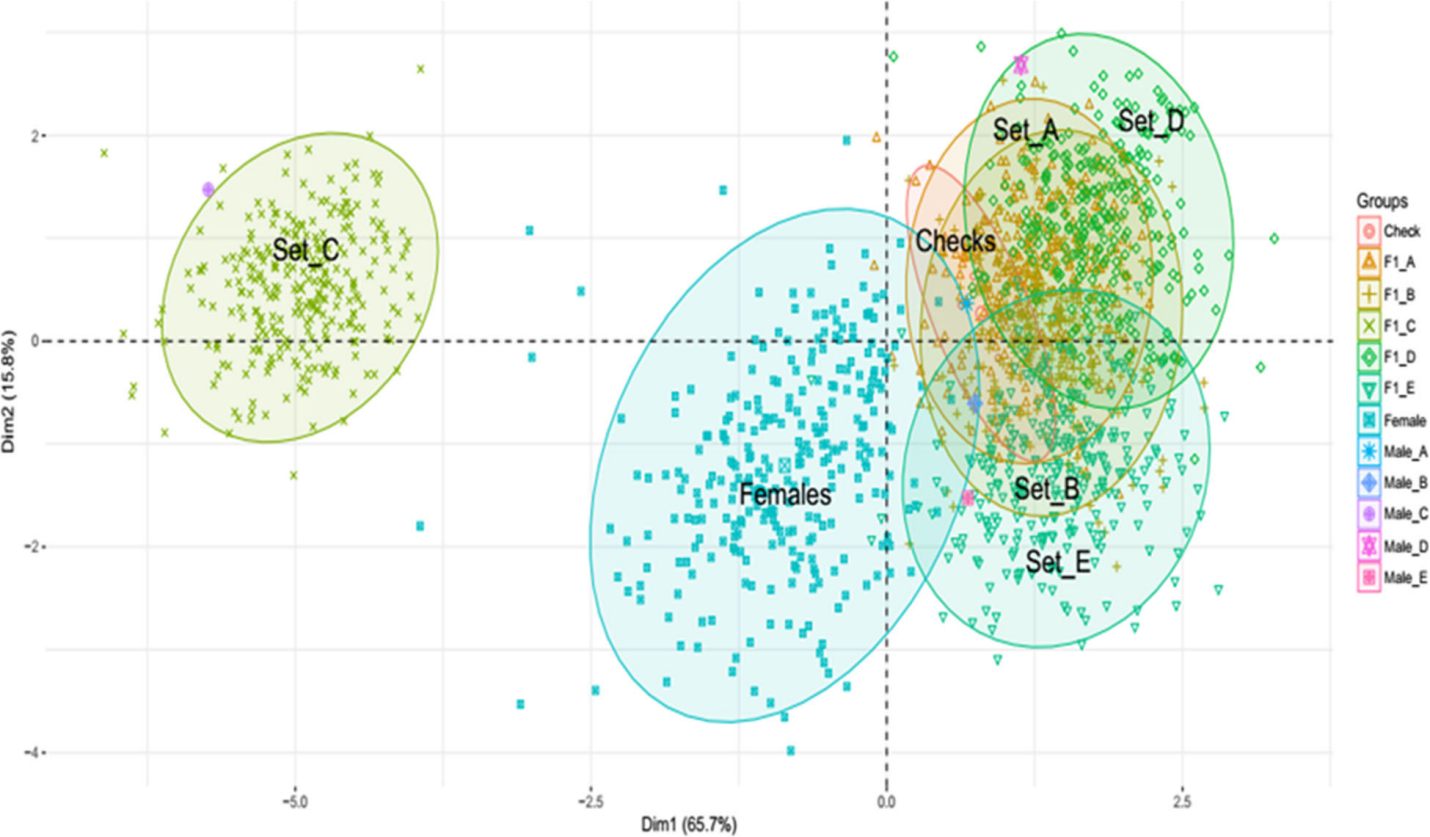
Scatter diagram of F_1_s and Parents in upland cottons based on phenological data projected in the (Dim1-Dim2) plane. Different colours depicting the distinct groups of lines, testers, F_1_s and checks. Abbreviations: Dim1., PC-1; Dim2., PC-2; A., 7886 tester; B., Zhong 1421 tester; C., A971 Bt tester; D., 4133 Bt tester; E., SGK 9708 tester

The scatter diagram of PCA for the studied material depicted a considerable amount of variability presence among lines, testers and F_1_s. First and second principal components (PC1 and PC2) of parents and F_1_ populations was plotted in which three major distinct groups were encountered including two main groups of F_1_s and one of female parents. Further details displayed five clusters of F_1_ populations according to their male parents (Fig. 2). Every sub-cluster of F_1_s is lying apart clearly indicating their diversity from each other. Furthermore, the presence of paternal parents alongside their respective F_1_s sub-clusters is validating the diversity. The second main cluster of F_1_ is representation of clear difference between hybrids originated from C tester and rest of hybrids from other testers.

**Fig. 3:**
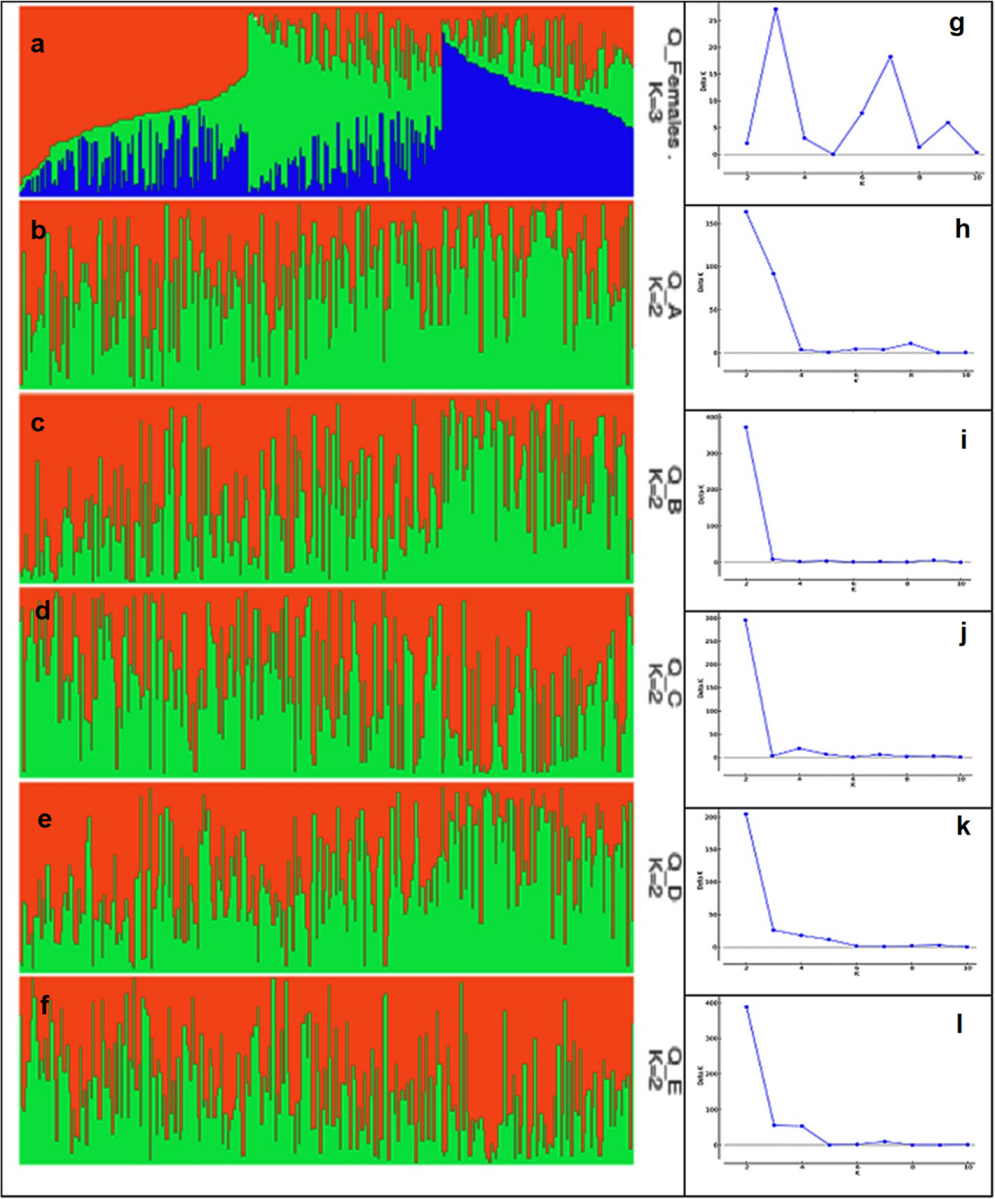
**a**, **b**, **c**, **d**, **e**, **f** The summary plots of Q-matrix estimates based on Bayesian posterior probability and Line charts of K with respect to SK for F_1_s from A, B, C, D and E male parents and 284 Female parents respectively. **g**, **h**, **i**, **j**, **k**, **l** SK values exhibited a maximum likelihood at K = 3 in Female parents (suggesting the total panel division into three subpopulations) while K = 2 in all the F_1_ hybrids

The LnP(D) values sustained to escalate without variation. Hence, K values could be determined with ∆K. The ∆K showed highest peak at K = 3, in case of Female parents while in all F_1_ hybrids, ∆K was maximum when K = 2, which suggested that the investigated material of female parents and hybrids might be distributed in three and two subdivisions respectively (Fig. 3). Figure 3 related to the population structure is showing a clear difference among the five sets of hybrids which laid the foundation for doing association analyses.

**Fig. 4:**
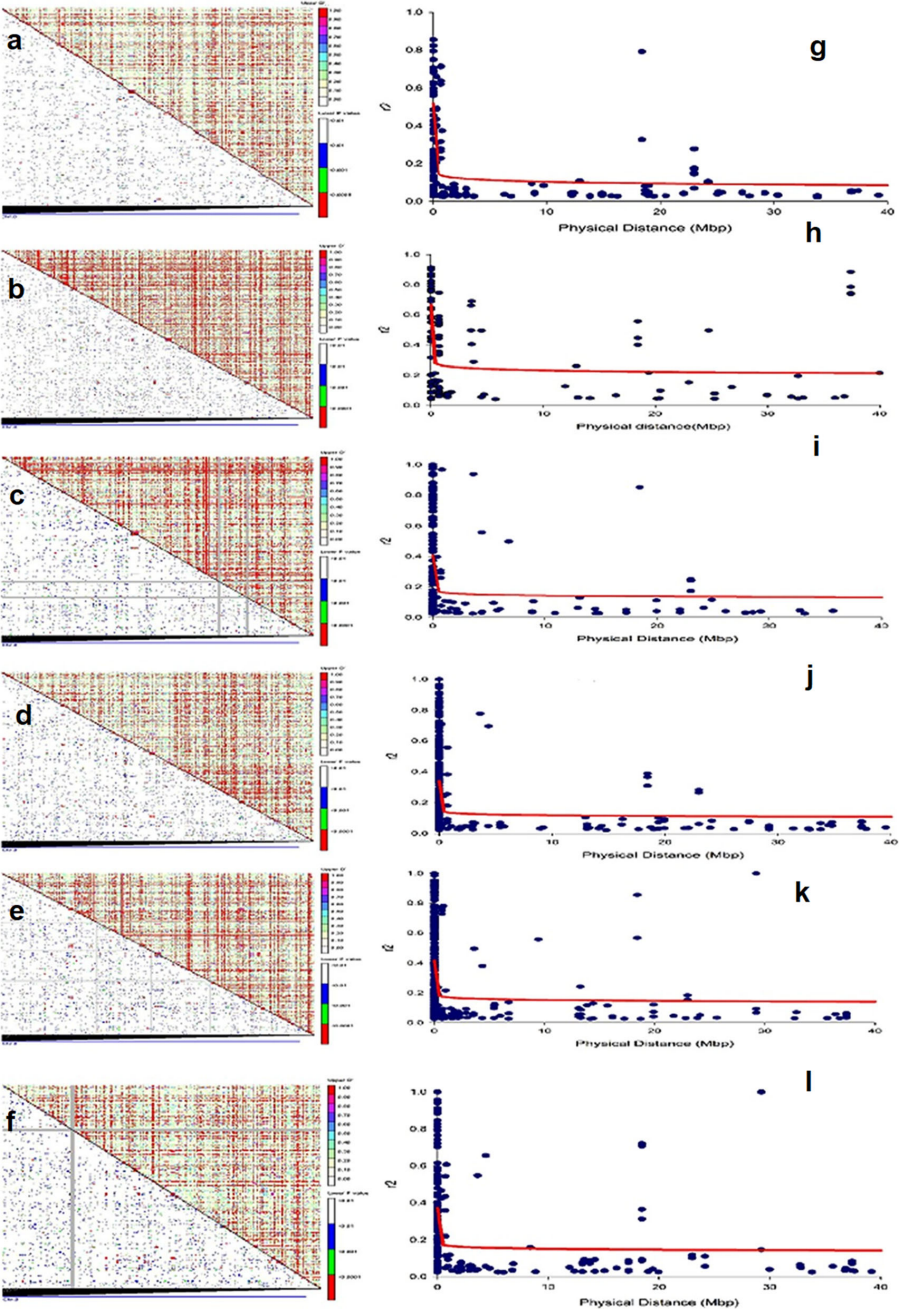
**a**, **b**, **c**, **d**, **e**, **f** Linkage disequilibrium distribution patterns between all possible loci pairs of female parents and F_1s_, Set-A, set-B, set-C, set-D, set-E respectively across various chromosomes. Each pixel on upper side of diagonal indicates size of *D′* related to corresponding marker pair as revealed with the *color code* at top right; whereas lower side of diagonal specifies *P* value of respective marker pair LD as revealed with the *color code* at the bottom right: white *p* > 0.05, blue 0.05 > *p* > 0.01, green 0.01 > *p* > 0.001 and red *p* < 0.001. **g**, **h**, **i**, **j**, **k**, **l** Scatterplots of the significant LD (*r*^*2*^) against physical distance (Mb) of female parents and F_1_ set-A, set-B, set-C, set-D, set-E respectively. The trend line (inner fitted) is a logarithmic regression curve based on *r*^*2*^ against physical distance

The association mapping based on LD was followed as described by Yu et al. in 2006 [30] using the TASSEL software package. The values of LD among all marker pairs have been plotted as LD plots to predict the LD patterns genome wide and estimate LD blocks. LD plots against physical map distance were generated in SigmaPlot 12.5 software, keeping r^2^ values with *P* < 0.001. The 0 cM r^2^ values were assumed as 0.0000001 following previous related reports [31]. The intra-chromosomal LD declined at physical distance ranging between 240-300kbp (r^2^ = 0.2) revealing the potential for association mapping (Fig. 4). The average linkage disequilibrium (LD) decay distance was 288kbp (r^2^ = 0.2).

### Marker-trait association studies

**Fig. 5:**
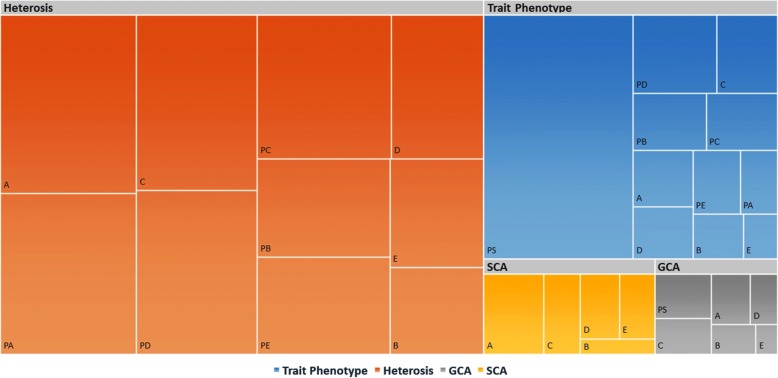
Summary of contributions delivered by dependent variables under study: trait phenotype, heterosis, specific combining ability (SCA) and General combining ability (GCA) for discovering significant (−log_10_ > 3) associations in L × T mating design. Size of each block is depiction of amount of significant associations in respective category of combinations. Abbreviations: A., Genotype & phenotype data of F_1_s from 7886 (A) tester; B., Genotype & phenotype data of F_1_s from Zhong 1421 (B) tester; C., Genotype & phenotype data of F_1_s from A971 Bt (C) tester; D., Genotype & phenotype data of F_1_s from 4133 Bt (D) tester; E., Genotype & phenotype data of F_1_s from SGK 9708 (E) tester; PA., Genotype data of maternal lines-phenotype data of F_1_s from 7886 (A) tester; PB., Genotype data of maternal lines-phenotype data of F_1_s from Zhong 1421 (B) tester; PC., Genotype data of maternal lines-phenotype data of F_1_s from A971 Bt (C) tester; PD., Genotype data of maternal lines-phenotype data of F_1_s from 4133 Bt (D) tester; PE., Genotype data of maternal lines-phenotype data of F_1_s from SGK 9708 (E) tester; PS., Genotype & phenotype data of Parents (Females)

Both Q matrix and kinship were integrated in the genetic model for association mapping following MLM using TASSEL software. Considering the results from all types of possible combinations (Parents, A, B, C, D, and E F_1_s sets) run through TASSEL, below probability α  =  0.001 (−log_10_  >  3) level. Collectively, 2846 associations were discovered at α  =  0.001 (−log_10_ >  3) related to four variables i.e., 787 associations with trait phenotype, 121 with GCA, 168 with SCA and 1770 with heterosis (Fig. 5). Out of them, 831 significant associations were detected between 176 microsatellites and 10 traits (Additional file 2). The description regarding 831 significant associations is given here as:

FL showed 75 significant associations with different microsatellites. Sixty-eight microsatellites displayed association with MIC. FS displayed 65 associations with microsatellites. BN showed association with 65 microsatellites from all the subsets. FUI depicted 65 significant associations with microsatellites. BW depicted association with 63 microsatellites. FE showed association with 60 microsatellites. Fifty-five significant associations have been displayed by FU with microsatellites. Fifty-five associations have been observed between PH and microsatellites. Fifty-four microsatellites showed association with LP (Additional file 3).

### Traits associated with microsatellites

**Fig. 6:**
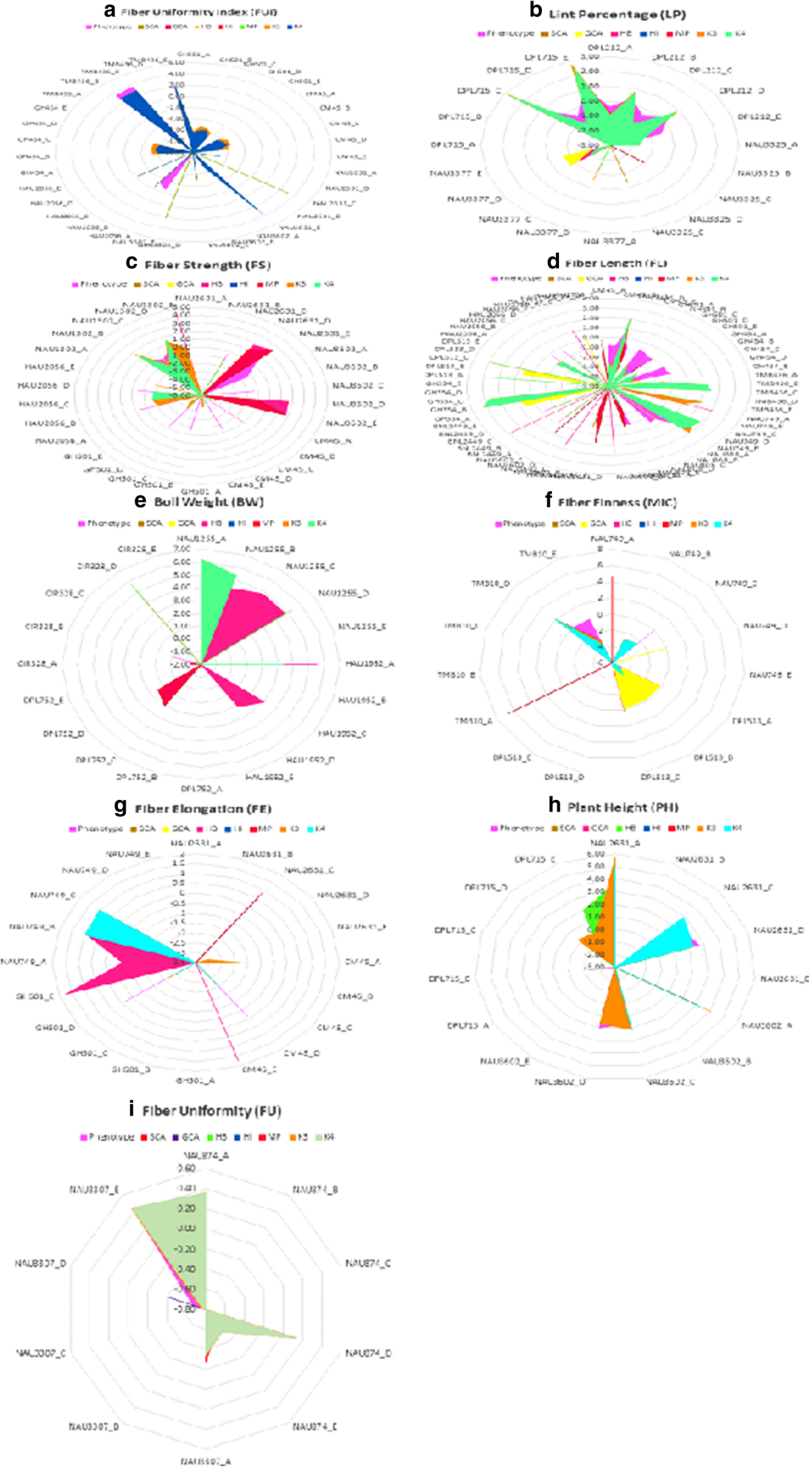
Significant associations (-log10>3) of (**a**) Fiber Uniformity Index (FUI), (**b**) Lint Percentage (LP), (**c**) Fiber Strength (FS), (**d**) Fiber Length (FL), (**e**) Boll Weight (BW), (**f**) Fiber Fineness (MIC), (**g**) Fiber Elongation (FE), (**h**) Plant Height (PH) and (**i**) Fiber Uniformity (FU) with microsatellites displaying their respective phenotypic effects. Color shading indicates an individual dependent variable that is Phenotype, SCA, GCA and Heterosis types. Abbreviations: A.,7886; B., Zhong 1421; C., A971 Bt; D., 4133 Bt; E., SGK 9708

A set of highly significant 46 microsatellites out of 176 loci found their associations with FUI, LP, FS, FL, BW, MIC, FE, PH and FU (Fig. 6). These loci were identified on the basis of their presence in trait phenotype, GCA, HB, MP and K4 in F_1_ hybrids descended from at least 3 testers (Additional file 4).

#### FUI

Seven QTLs were identified for FUI based on trait phenotype, HB, HI, MP, K3 and K4. This trait has been found to be associated with following microsatellites: GH454, CM45, GH501, HAU2056, NAU2631, NAU3602 and TMB436. These QTLs have been identified with dominance effects except FUI_HAU2056 (F_1_ from A tester) with additive effect (Additional file 4).

#### LP

Total 4 QTLs were detected on the basis of trait phenotype, GCA, SCA, HB, HI, MP, K3 and K4. This trait has shown association with microsatellites; DPL715, DPL212, NAU3325, NAU3377 representing dominance effects and one QTL; LP_NAU3377 (F_1_ from B tester) with additive effect (Additional file 4).

#### FS

In total, 6 microsatellites have been detected in close association with FS. The QTL associated with NAU2631 was detected with dominance effects based on trait phenotype, HB, HI, MP, K3 and K4 while only one was with additive effect from F_1_ of A-tester. The QTL associated with NAU3602 was identified with additive (F_1_ from A-tester) and dominance effects on the basis of trait phenotype, HB, HI, MP, K3 and K4. QTL linked with CM45 was identified based on trait phenotype, K3 and K4 with additive (F_1_ from B-tester) and dominance effects. QTL linked with GH501 was identified based on trait phenotype, K3 and K4 with additive (F_1_ from B-testers) and dominance effects. QTL linked with HAU2056 was identified based on trait phenotype, K3 and K4 with dominance effects. QTL linked with NAU1302 was identified based on trait phenotype, GCA, HB, HI, MP, K3 and K4 with dominance effects (Additional file 4).

#### FL

A total of 13 microsatellites have been identified in association with FL. The QTLs associated with CM45 and GH501 were discovered with dominance effects based on trait phenotype, HI, MP, K3 and K4. The QTLs distinguished with GH454, TMB436 and HAU2056 were detected with dominance effects based on trait phenotype, K3 and K4. The QTL linked with NAU749 and GH354 was detected with additive (F_1_s from D and A tester respectively) and dominance effects based on trait phenotype, GCA, HB, K3 and K4. QTL associated with NAU808 was identified with additive (F_1_s from A and B tester) and dominance effects based on trait phenotype, GCA, HB, MP and K4. The QTLs associated with NAU2631 and NAU3602 were discovered with dominance effects based on trait phenotype, SCA, HB, HI, MP, K3 and K4. The QTLs associated with BNL2449 was discovered with dominance effects based on trait phenotype, HB, K3 and K4. The QTL linked with DPL513 was detected with additive (F_1_s from B and C tester) and dominance effects based on trait phenotype, GCA, K3 and K4. The QTL associated with HAU2759 was noticed with additive (F_1_ from C tester) and dominance effects based on SCA, GCA, HB, HI and MP (Additional file 4).

#### BW

Total 4 QTLs have been identified for BW. The QTL discovered with NAU1255 exhibited dominance effects based on trait phenotype, HB, K3 and K4. The QTLs associated with HAU1952 was discovered with dominance effects based on trait phenotype, GCA, HB, K3 and K4. The QTL discovered with DPL752 displayed dominance effects based on HI and MP. The QTL associated with CIR328 was observed with dominance effects based on trait phenotype, GCA, HB, K3 and K4 (Additional file 4).

#### MIC

Three QTLs for MIC have been identified. The QTL associated with NAU749 was identified based on trait phenotype, GCA, HI, MP, K3 and K4 with dominance and additive (F_1_ from D tester) effects. The QTL related with DPL513 was identified based on trait phenotype, SCA, GCA, HB, K3 and K4 with dominance and additive (F_1_ from B tester) effects. The QTL related with TMB10 was identified based on trait phenotype, HB, HI, MP, K3 and K4 with dominance effects (Additional file 4).

#### FE

Total 4 QTLs for FE have been discovered. The QTL associated with NAU2631 was identified based on HB, HI, MP, K3 and K4 with dominance effects. The QTLs associated with CM45, GH501 and NAU749 were identified based on trait phenotype, HB, K3 and K4 with dominance effects (Additional file 4).

#### PH

Total 3 QTLs for PH have been discovered. The QTLs associated with NAU2631 and NAU3602 were identified based on trait phenotype, HB, HI, MP, K3 and K4 with dominance effects. The QTL associated with DPL715 was identified based on trait phenotype, GCA, HB, K3 and K4 with dominance and additive (F_1_ from B and E tester) effects (Additional file 4).

#### FU

Two QTLs have discovered for FU. The QTL associated with NAU874 was identified based on trait phenotype, SCA, HB, HI, MP, K3 and K4 with dominance effects. The QTL linked with NAU3307 was identified based on trait phenotype, SCA, GCA, K3 and K4 with dominance and additive (F_1_ from D tester) effects (Additional file 4).

These QTLs were detected based on being appeared in F_1_s from at least 3 out of five testers, each with a different dependent variable. Noticeably, every type of effect was identified with trait phenotype, dominance effects were found with SCA, HB, HI, MP, K3 and K4 while additive effects were identified with GCA. Although in above results the dominance effect of few QTLs have been detected with GCA but their effect was close to zero. The main purpose of this experiment was to work out the comparison among genetic components of above mentioned four dependent variables and to verify the presence of detected highly associated QTLs in the hybrids of five testers which were screened for ten agronomic and fiber quality related traits at various locations for 2 years.

**Fig. 7:**
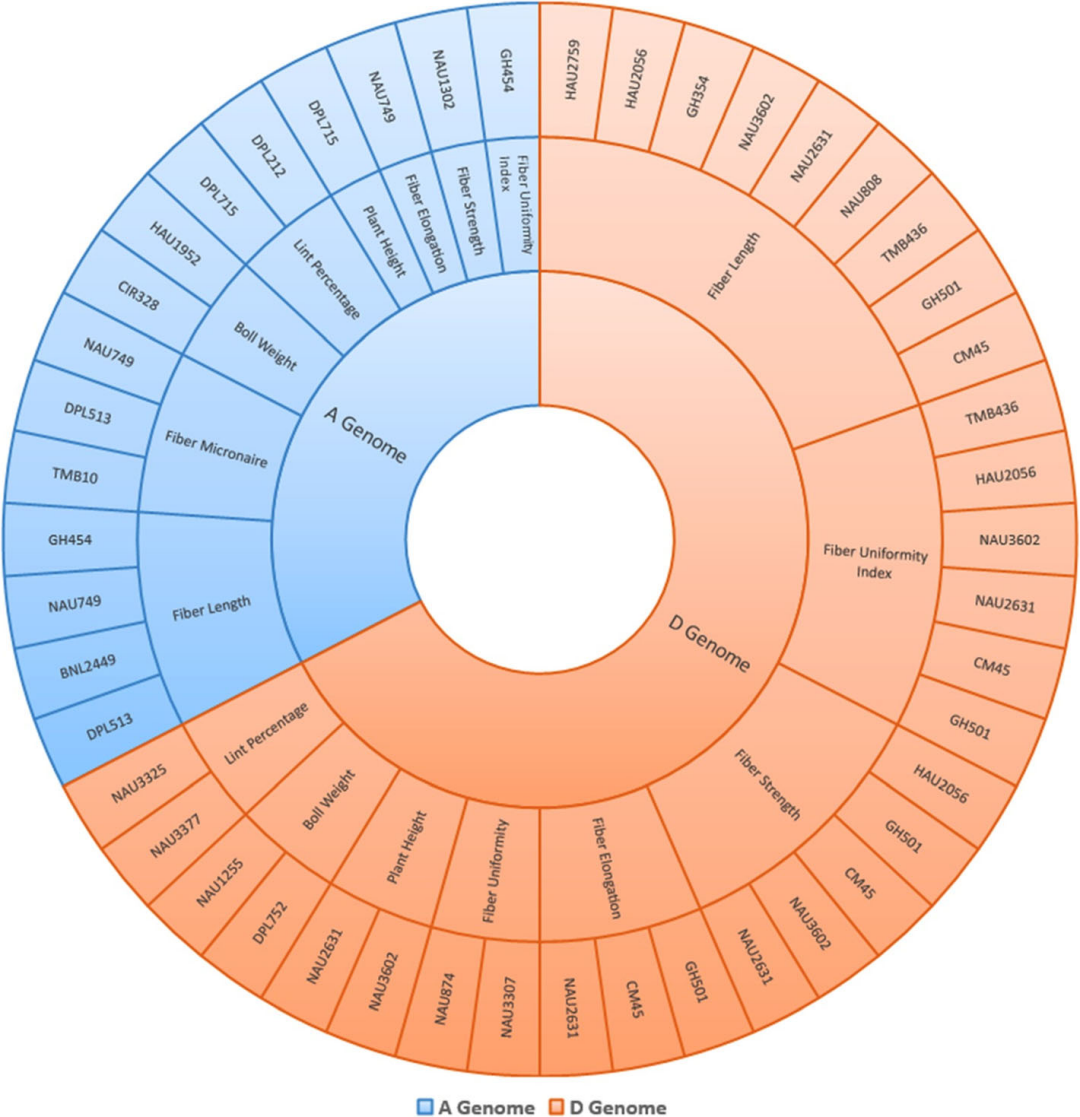
Summary of significantly (*p* < 0.001) associated microsatellites with phenotypic traits based on their distribution on A and D sub-genomes. Eight phenotypic traits found their significant associations with 15 microsatellites distributed on A sub-genome and 8 phenotypic traits got significant associations with 31 microsatellites from D sub-genome

It was observed that two-thirds of the highly significant (*p* < 0.001) associated microsatellites showed their presence on D sub-genome, especially those of FS, FL and FU. Also the pleiotropic effects of loci NAU2631, CM45 and GH501on phenotypic traits FUI, FS, FL and FE were discovered (Fig. 7).

**Fig. 8:**
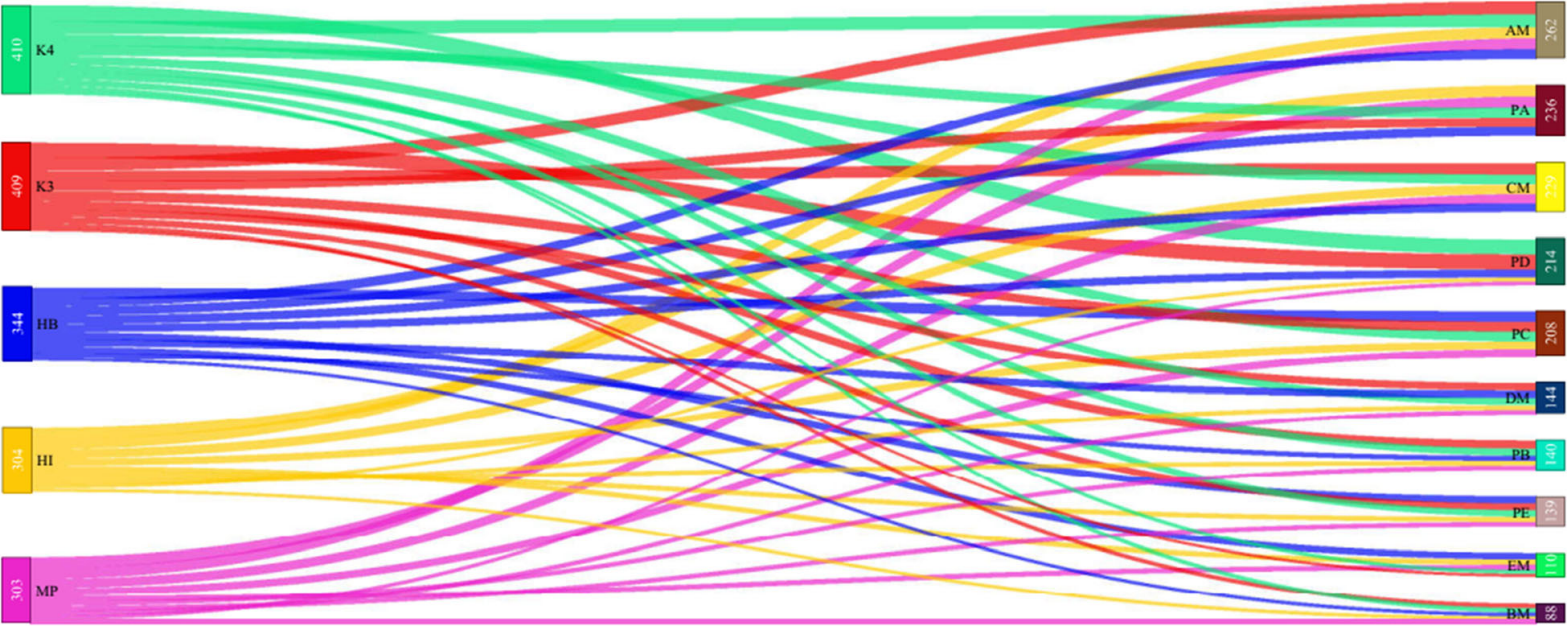
Power for detection of hQTLs in significant (−log_10_ > 3) associations ranked according to amount of associations detected. Viscosity of each originating link is indicating the power of hQTL detection in terms of association numbers. Abbreviations: HB., Heterobeltosis; HI., Heterosis Index; MP., Mid-Parent Heterosis; K3., Heterosis over Check K3; K4., Heterosis over Check K4; AM., Genotype & phenotype data of F_1_s from 7886 (A) tester; BM., Genotype & phenotype data of F_1_s from Zhong 1421 (B) tester; CM., Genotype & phenotype data of F_1_s from A971 Bt (C) tester; DM., Genotype & phenotype data of F_1_s from 4133 Bt (D) tester; EM., Genotype & phenotype data of F_1_s from SGK 9708 (E) tester; PA., Genotype data of maternal lines & phenotype data of F_1_s from 7886 (A) tester; PB., Genotype data of maternal lines & phenotype data of F_1_s from Zhong 1421 (B) tester; PC., Genotype data of maternal lines & phenotype data of F_1_s from A971 Bt (C) tester; PD., Genotype data of maternal lines & phenotype data of F_1_s from 4133 Bt (D) tester; PE., Genotype data of maternal lines & phenotype data of F_1_s from SGK 9708 (E) tester; PS., Genotype & phenotype data of maternal lines

From five types of heterosis and respective 10 different possible, combinations used in the association analysis specifically for analyzing heterosis, a whole sum of 1770 significant (−log_10_ > 3) associations have been identified. The detail is given here as: from HB 344 associations, from HI 304 significant associations, from MP heterosis 303, from heterosis over check-K3 409 and heterosis over check-K4 410 significant associations have been discovered (Fig. 8). Newly discovered heterosis quantitative trait locus (hQTLs) including 7, 1, 3, 9, 3, 1, 3, 3 and 2 loci for FUI, LP, FS, FL, BW, MIC, FE, PH and FU respectively are one of prominent findings from current study.

### Discovery of favorable alleles

**Fig. 9:**
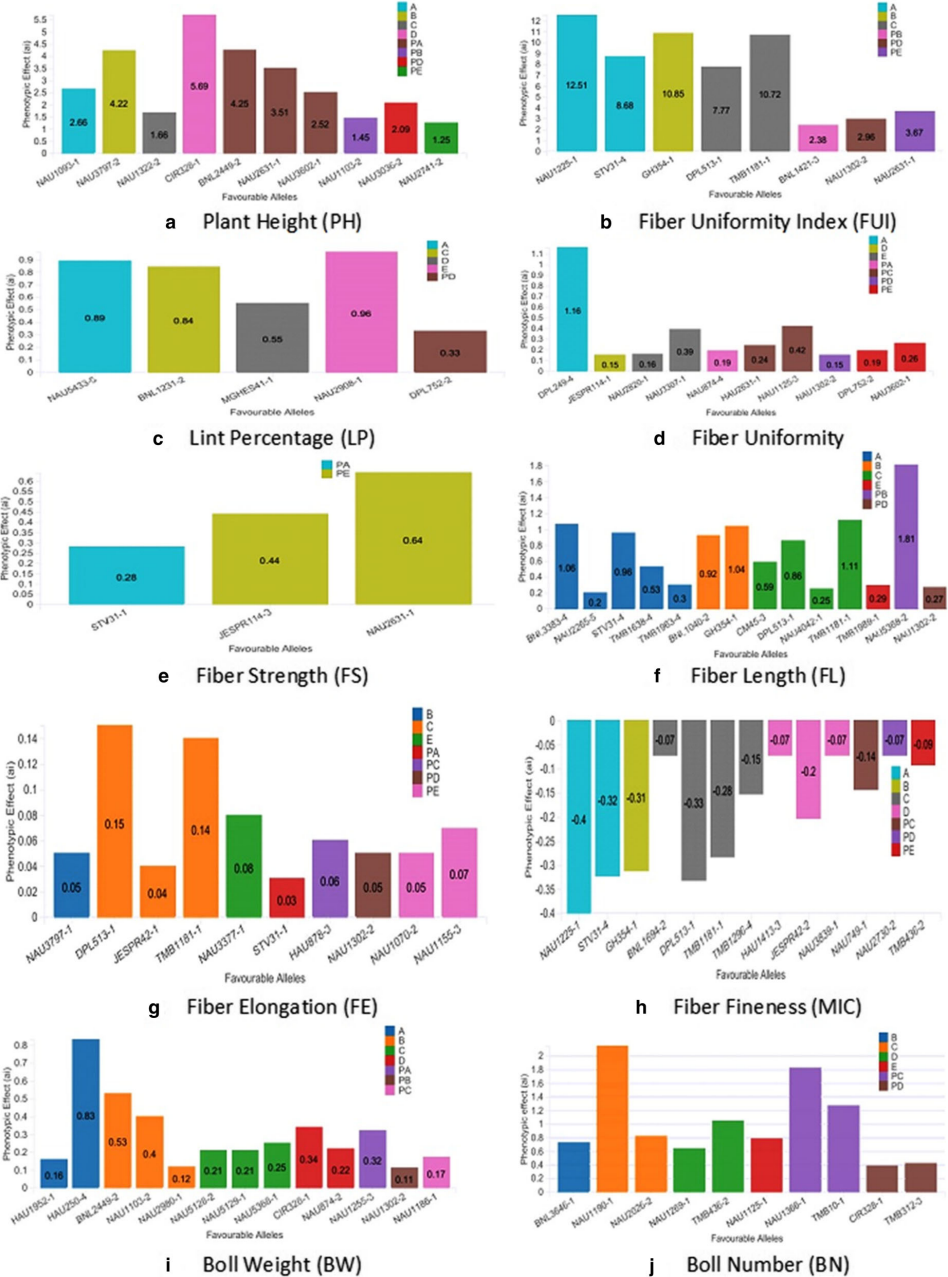
Favorable alleles of significant (-log10>3) QTLs for (**a**) Plant Height (PH), (**b**) Fiber Uniformity Index (FUI), (**c**) Lint Percentage (LP), (**d**) Fiber Uniformity (FU), (**e**) Fiber Strength (FS), (**f**) Fiber Length (FL), (**g**) Fiber Elongation (FE), (**h**) Fiber Fineness (MIC), (**i**) Boll Weight (BW), (**j**) Boll Number (BN) with their respective phenotypic effects (ai). Representative combinations of phenotype and genotype data used in TASSEL association analysis with abbreviation: A., Genotype & phenotype data of F1s from 7886 tester; B., Genotype & phenotype data of F1s from Zhong 1421 tester; C., Genotype & phenotype data of F1s from A971 Bt tester; D., Genotype & phenotype data of F1s from 4133 Bt tester; E., Genotype & phenotype data of F1s from SGK 9708 tester; PA., Genotype data of maternal lines-phenotype data of F1s from 7886 tester; PB., Genotype data of maternal lines-phenotype data of F1s from Zhong 1421 (B) tester; PC., Genotype data of maternal lines-phenotype data of F1s from A971 Bt tester, PD., Genotype data of maternal lines-phenotype data of F1s from 4133 Bt (D) tester; PE., Genotype data of maternal lines-phenotype data of F1s from SGK 9708 tester

Phenotypic effects of each significantly (−log_10_ > 3) identified QTLs were estimated with maximum positive and minimum negative allele effects in all environments and all possible combination of phenotype and genotype data used in running of TASSEL association analysis for superior lines, testers and F_1_s (Fig. 9).

According to BLUP results obtained from association analysis, 831 significantly associated (−log_10_ > 3) loci genotype data found their association with 10 traits phenotype data at 10 locations for two tears and 96 elite alleles were discovered from them. At -log_10_ > 3 level, 96 substantial associations were discovered between microsatellites and phenotypic parameters regarding superior alleles effects. The superior alleles have been recognized based on breeding objective related to each target trait. Based on mentioned procedure, the allele of significantly identified stable QTLs (−log_10_ > 3) have been evaluated regarding their respective phenotypic effects. Most prominently the combination of phenotype and genotype data taken from F_1_s of C tester contributed significantly in detecting superior alleles. Among detected superior alleles from this combination, TMB1181–1 depicted maximum positive phenotypic effects for FUI so increased FUI by 10.22%. However, DPL513–1 displayed minimum negative phenotypic effect for MIC so increased it by − 0.33. A range of 10.72 to − 0.33 has been estimated in this combination of phenotypic effects influencing BN, BW, FUI, FL, FE, LP, MIC and PH.

## Discussion

Earlier the scientists did not use heterosis concept for self-pollinated crops due to lack of hybrid vigor and other related theories. Afterwards, scientists of recent decades utilized the idea of heterosis in rice for the improvement of yield and related queries and ultimately obtained fruitful results. Getting inspiration from this breakthrough, we tried to exploit the concept by integrating conventional and advanced molecular tools to clarify and validate the mechanisms involved in heterosis, which is hardly utilized by earlier cotton breeders. We have used F_1_ hybrids in L × T mating design instead of segregating populations (bi-parental crossing) for the sake of dissecting genetic foundation of heterosis and detected different types of QTLs via GWA mapping; related to trait phenotype, GCA, SCA, HB, HI, MP, K3 and K4. Such type of information is merely available previously, as very few studies have been conducted to explain the basis of genetics involved in heterosis in cotton. A QTL mapping strategy has been approached in the current study, earlier proposed by Wen et al. in 2015 [32] to explain the main effects considered in single genetic model.

The correlation coefficients for most of the traits showed positive and significant correlation so these traits can be proved together with each other. However, the traits with significant negative correlation depicting the inverse relationship can be treated reverse for their improving. The scatter diagram and density distribution showed normal distribution of hybrids as well as parents. Therefore, the populations can be used for further analyses of corresponding traits without transformation. Though trait phenotype performed as best variable to genetically dissect basis of quantitative parameters as well as heterosis. Others are helpful for estimating main effects as GCA and trait phenotype are suggested for identifying additive effects, while SCA along with trait phenotype for distinguishing dominance effects.

L × T is an efficient parental mating design to study combining ability and heterosis. Also it is utilized to evaluate the genetics of different traits and their variance [33]. It aided estimation of gene effects of quantitative traits [34] in different crops like maize, rice and cotton.

The additive QTLs are more powerfully detected with GCA rather than trait phenotype, which is confirmed by MIC_NAU749, MIC_DPL513, LP_NAU3377, FL_NAU749, FL_NAU808, FL_DPL513, FL_HAU2759, FL_GH354, FU_NAU3307 and PH_DPL715 additive QTLs. However, SCA had comparatively lesser power than trait phenotype, and heterosis had a bit lesser power than SCA in distinguishing dominance related QTLs. The proposed method delivers options in the genetic dissection of heterosis, which can further be utilized to confirm the outcomes.

Many previous studies have found different QTLs related to fiber yield and quality concerned parameters [35–42]. However, it is hard to relate the QTLs identified in these studies because few common markers occurred in the miscellaneous populations employed. Also the maps shaped in these studies harbored different chromosome regions of cotton genome. Both previous and present studies have shown many common featured QTLs mapped to the same chromosomes. We compared our results with those reported in different publications on F_2_ populations from different inter and intraspecific crosses though different types of population (F_2_, RI, BCRI, BCF_2_ etc) were employed.

BW has been discovered to be associated (*p* < 0.001) with CIR328 [43, 44], FE with NAU749 [45], FL with BNL2449 [43, 44], HAU2759, NAU749 [45] and TMB436 [46], FS with HAU2056 [45], NAU1302 [47, 48], NAU2631 [35], FUI with TMB436 [46], LP with DPL212, NAU3377 [42, 45] and DPL715 [46] and MIC with NAU749 and TMB10 [45]. Remaining hQTL associations have been discovered as novel findings.

As a consequence, comparing phenotypic values associated with superior alleles for each target trait, we dissected 22, 19, 19, 23, 7, 16, 12, 8, 22 and 18 favorable alleles for BN, BW, FE, FL, FS, FU, FUI, LP, MIC and PH respectively. After bird’s eye view, investigation of association results depicted that female lines contributed a lot in mining of superior alleles. We suggest the use of this tester primarily for the introgression of superior alleles that got transferred from founder parents. These influential superior alleles from this specific combination is provision of the fact that A971 Bt (C) tester is great potential harbored cotton cultivar of China. It should beneficially be used in advance breeding programs aimed at exploitation of hybrid vigor.

With the passage of time, climatic changes pose threats to crops in the lane of their successful survival. Whereas crops genetic banks lack much diversity to cope with situations due to limited founder parents and so with upland cottons of China. Keeping in view the scenario, its urgent need of time to go for thorough search of the genetic variations that may have emerged and amassed in genetic banks of cotton cultivars during their breeding history in order to exploit them for the introduction of additional diversity platforms to triumph wider genetic base.

For the improvement of complicated traits, of course molecular techniques including primarily the associated QTLs of fibre related features are of prime importance but the less time consuming and reliable tactic lies in the development and use of F_1_ generation in breeding programs. Genome wide studies are authenticating the reliability of using F_1_ individuals by providing scientific grounds to mine, conserve and efficiently exploit favorable QTLs that are of our interest.

In current era, via whole genome sequencing of *G. hirsutum* an SNP chip NAUSNP80K, has been developed fruitfully that can be efficaciously utilized to perform cotton GWAS. Hence, utilization of SNP in huge mass for backing up GWAS in cotton will be our further pace in advanced cotton realm that would definitely provide sound basis for provision of information connected to protein coding genetic factors via exploitation of bioinformatics tools and transgenics of quantitative factors. Consequently, improvements in cotton yields are just, combination of computer simulations with breeding programs away.

## Conclusions

Highly significant 46 microsatellites were discovered in association with FUI, LP, FS, FL, BW, MIC, FE, PH and FU. Two-thirds of these significantly associated loci were scattered on D sub-genome, especially those of related to FS, FL and FU. Also the pleiotropic effects of NAU2631, CM45 and GH501 loci on FUI, FS, FL and FE were detected. A set of 96 exclusively favorable alleles were discovered primarily associated with BW, FL, FE and MIC mainly harbored by F_1_s from C tester (A971 Bt). To grab prominent improvement in mentioned influenced fiber quality and yield traits, we suggest the A971 Bt cotton cultivar as fundamental element in succeeding AM population development procedure to eliminate deleterious alleles residing at corresponding loci of superior alleles. The output of this study can be helpful for plant breeders and researchers working to improve the yield and quality attributes of cotton for the efficient utilization of hybrid vigor.

## Methods

### Association mapping panel construction

A total collection of 284 exclusive upland cotton pure lines from gene bank of ICR (Institute of Cotton Research), CAAS (Chinese Academy of Agricultural Sciences) and renowned top 5 cultivars from different regions of China as testers were efficiently utilized for current experimental study. Among these accessions, 238 (83.8%) were collected from diverse cotton growing areas including yellow river valley, Yangtze river valley and Northern area in China. The remaining 46 (16.2%) were introduced from 11 different countries (USA, Russia, Australia, Burundi, Chad, Ivory Coast, Kenya, Sudan, Turkmenistan, Uganda, and Vietnam). These accessions have been planned to utilize on the basis of their improved agronomic and fiber related features supremely fiber quality, fiber yield, fiber maturity, boll number, boll size and both abiotic and biotic stress resistances [49].

### Mating design

Here in this study, Line × Tester (L × T) mating design has been utilized. This design was suggested by Kempthorne for the first time in 1957 [14]. This design implicates hybridization among female lines and testers in one to one fashion for production of hybrids [33]. It gives SCA as well as GCA of every cross for lines and testers respectively [33]. In addition, it provides estimation of gene actions related to different types that prove significant in the expression of metric traits [34].

### Field planting and traits examination

Field plantation of experimental material was conducted in cotton growing seasons in 2012–2013 at different locations of China cotton belt mainly covering Yangtze River and Yellow River regions. The locations include Anyang (AN), Baoding (BD), Dongying (DY), Hejian (HJ) and Xinxiang (XX) from Yellow River region, while Changsha (CS), Changde (CD), Jiujiang (JJ), Wuhan (WH) and Jingzhou (JZ) in Yangtze River region. There exists a variation in agro-ecological features in different growing regions considered i.e.; climate and cotton management practices considering primarily soil fertility, precipitation amount, temperature, growing period and agronomic practices [50].

High yielding accessions from primary gene pool of upland cotton (*G. hirsutum*) were selected as male and female parents. Two hundred eighty-four female parents were mated with 5 male parents namely 7886 (A tester), Zhong 1421 (B tester), A971 Bt (C tester) 4133 Bt (D tester) and SGK 9708 (E tester) in proper pattern to produce F_1_ hybrid population. Field trials of the F_1_ populations and parents were conducted at ten different locations for 2 years. Field experiments followed a randomized complete blocked design with three replications at each location. F_1_ population from five groups (A, B, C, D and E) and 284 female parental lines were grown at ten different locations for 2 years. Ten yield and fiber quality related traits viz. plant height (PH), boll weight (BW), lint percentage (LP), bolls per plant (BN), upper half mean length (FL), fiber strength (FS), micronaire (MIC), fiber uniformity (FU), fiber elongation (FE) and fiber uniformity index (FUI) were recorded from each set containing F1 s and female parents from all locations. Data collection related to yield related characters was done after randomly selected and tagged 10 guarded individual plants. After attaining 70% of boll opening, 3 bolls per tagged individual plants (from middle branches) from each plot were harvested and estimated for seed cotton yield and related traits. About 150 g of lint samples from ginned samples bolls with roller gin for examining fiber-associated features. Fiber quality data was scored with high volume instrument (HVI) in the Laboratory of Quality & Safety Risk Assessment for Cotton Products (Anyang), Ministry of Agriculture, People’s Republic of China.

Five types of Heterosis viz.; Heterobeltosis (HB), Heterosis index (HI), Mid-Parent heterosis (MP) and standard heterosis using two commercial Chinese cotton cultivars i.e., Rui za 816 (K3) and Eza mian 10hao (Tai D5) (K4) and both kinds of combining abilities (general and specific) were estimated.

### DNA isolation and microsatellites fingerprinting

Molecular markers of simple sequence repeats type were surveyed on experimental material in an amount of 203 with high polymorphism. These were from diverse series including BNL, CIR, CM, DPL, GH, HAU, JESPR, MGHES, MUCS, MUSS, NAU, STV and TMB. CottonGen and Cotton Marker Database were searched for sequences of mentioned microsatellites. These markers were uniformly distributed all over the 26 chromosomes of cotton with an approximate average of 7.6 marker/chromosome.

Young leaves (2–3) from randomly selected plants were sampled for DNA extraction and stored at − 70 °C. CTAB method [51] was used for extraction of genomic DNA from young leaves of every genotype. Quality of DNA was then assessed on 1% agarose gel via electrophoresis.

The protocols of PCR cocktail preparation, amplification and electrophoresis all were followed as set by Zhang and Stewart in 2000 [34]. PCR reaction mixture was prepared with a total volume of 10 μL comprising 1.2 μL DNA (50 ng/μL), 0.2 μL *Taq* DNA polymerase (2 U/ μL), 0.2 μL dNTP mix(10 mM), 0.65 μL (5 μM) each for forward and reverse primer pair, 1 μL 10× PCR buffer (20 mM Mg^+ 2^ and 6.1 μL ddH_2_O. Thermal cycler conditions set for reaction were as follows: 3 min of initial denaturation at 95 °C, 30s for 30 cycles of denaturation at 95 °C, 50s for both annealing at 57 °C and extension at 72 °C and 7 min of final extension again at 72 °C. After completion of every PCR the samples were hold at 4 °C.

Electrophoresis was performed by using 8% PAGE in 1× TBE electrolytic solution to visualize the PCR amplified products. Electrophoretic apparatus comprised vertically loaded gel on both sides each having 96 comb lane. For estimation of amplified DNA products size a 50 bp ladder was kept as standard. Silver staining was performed to visualize bands whilst UV light board was used to read and record bands sizes. Amplified band of every microsatellite locus was recorded in binary form as ‘0’ for absence and ‘1’ for presence of band.

### Phenotypic data analysis

Morphological data of fiber-associated attributes especially yield and quality, were taken from 284 lines, 5 testers and 284 respective F_1_s from each cross at each location for consecutive 2 years of study and summary statistics was workout and further subjected to ANOVA for RCBD [52].

For classical multivariate techniques, covariance and correlation matrices (together with mean vectors) provide enough statistics with sound basis of multivariate normal linear models. For the analysis of multivariate structure, various tools with statistical background are at hand which mainly include canonical correlation analysis, factor analysis, principal component analysis and so forth. In order to readily apprehend the relationship among variables with the main purpose of reducing the number of dimensions connected to their multivariate structure, the above mentioned tools are primarily utilized. Besides these, for the revelation of variables relationship among themselves some visualization practices for dimension-reduction have been additionally established which supremely take into account canonical structure plots [53], factor pattern plots, biplots [54] and so on. For enhanced simpler views of relationship among variables use of dynamic graphics on the basis of linear combinations and projections is another advanced technique encountering grand tours [55] and exploratory projection-pursuit [56]. Unfortunately, directly from correlation matrices for the interpretation of variables relationship among themselves fewer techniques are available. However, scatterplot matrix is an exceptional tool to visualize the variables relationship provided relatively less quantity of variables are required to scrutinize. It exhibits all the data and substantially enhance the representation by decorating it with regression lines (linear), (loess) smoothed curves, data ellipses and so on. Predominantly with non-parametric smooth curve, it becomes possible to define the variables relationships from scatterplot visualization whether linear or if some transformations would be useful. Onward to this, mostly it is assumed that all such similar complications have been dealt with along with consideration that all variables are linearly correlated with each other on some transformations scales. It develops some glitches in the direct display of data when we go beyond the limits of comparatively lesser variables data. Above discussed approach has been established for dimension-reduction sort of complications.

To possibly display the patterns of correlation among variables present in larger data set form, we pondered on techniques which can apprehend mentioned scenario of data. To attain this in logical manner, while dealing with relatively greater amount of variables an effective visual thinning (schematic visual summary) approach was utilized like in boxplot [57], that reduces details in the middle in order to depict more significant statistics on univariate shape, center, spread and outliers. The eigenvalues of the first two principal components and correlation coefficients were extracted for each genotype (F_1_s and parents) and their studied traits by using R software package.

### Evaluation of heterosis and combining ability

The percent increase or decrease of F_1_ hybrids over parent values were calculated using the formulas proposed by Fehr in 1987 [58] to estimate possible heterotic effects of the traits measured in the current study. The GCA variance of parents and SCA variance of hybrids were evaluated by following Line × Tester variance analysis as reported by Singh and Chaudhary in 1977 [59].

### Genotypic data analysis

#### Population structure

The Bayesian model-based program STRUCTURE 2.3.4 has been utilized to evaluate the population structure. The length of burn-in period and the number of Markov Chain Monte Carlo (MCMC) replications following burn-in were set at 100,000 having an admixture and allele frequencies correlated model. Ten independent run iterations were executed set with the hypothetical number of subpopulations (K) extending from 1 to 11. However, the outcomes represented a continuously increasing value of K with corresponding LnP(D) value. By integrating the probability data from [LnP(D)] obtained via STRUCTURE with ΔK (ad hoc statistic), K value was precisely estimated [60]. On the basis of this precise K, every genotype was given to the relevant subpopulation with membership value (Q value) > 0.5 [61], and so Q-matrix (population structure) was created for further association mapping of marker and traits. For the STRUCTURE software, “1” was used for fragments presence, “0” for fragment absence, and “-9” for missing data.

#### Association analysis and superior allele identification

To estimate LD pattern in Upland cotton genome, the weighted average of squared correlation coefficient r^2^ of each pair of microsatellites was calculated using the software package TASSEL 2.1 based on rapid permutations in 1000 shuffles with rare alleles (allele frequency less than 0.05) treated as missing data [31]. Every loci pair was ranked as linked or unlinked with the basis regarding their presence on same or different chromosome respectively. For both types of linked and unlinked markers LD was calculated in parental populations and hybrid populations taken from STRUCTURE analysis. The 99th percentile of r^2^ distribution for unlinked markers, which determined whether LD is due to physical linkage, was treated as the background LD level [62]. The r^2^ values of each pair of microsatellites were plotted against map distance (Mbp), and LD decay was estimated. By utilizing Sigmaplot version 12.5 an inner fitted trend line i.e., nonlinear logarithmic regression curve was sketched in order to elaborate the affiliation between r^2^ and Mbp of microsatellites prevailing on single chromosome.

Mixed linear model (MLM) was used to construct markers-fiber quality trait association tests using the TASSEL 2.0.1 software package [31]. For the TASSEL software, “1” designates presence of fragments, “0” specifies absence, and “?” designates missing value. The MLM association test was performed by considering Q-matrix and K-matrix simultaneously as followed by Yu et al. in 2006 [30]. False positive associations are significantly reduced by MLM model by considering the effects of both kinship and structure related to the material under investigation [30] and gives P and r^2^ values of each significant association. The detail of genotypic and phenotypic data combinations used in the TASSEL analysis is given in Table 2.

**Table 2:** Thirty-two combinations of genotype and phenotype data used in 4 sets of variables namely Traits Phenotype, Heterosis, GCA and SCA for running of TASSEL software

Sr. No.	Combinations	Genotype data	Phenotype data	Variable
1	1	PS	A	Trait Phenotype
2	2	PS	B	
3	3	PS	C	
4	4	PS	D	
5	5	PS	E	
6	6	A	A	
7	7	B	B	
8	8	C	C	
9	9	D	D	
10	10	E	E	
11	11	PS	PS	
12	1	PS	PS	GCA
13	2	A	A	
14	3	B	B	
15	4	C	C	
16	5	D	D	
17	6	E	E	
18	1	A	A	SCA
19	2	B	B	
20	3	C	C	
21	4	D	D	
22	5	E	E	
23	1	PS	A	Heterosis
24	2	PS	B	
25	3	PS	C	
26	4	PS	D	
27	5	PS	E	
28	6	A	A	
29	7	B	B	
30	8	C	C	
31	9	D	D	
32	10	E	E	

Note: A., Data of F_1_s from 7886 (A) tester; B., Data of F_1_s from Zhong 1421 (B) tester; C., Data of F_1_s from A971 Bt (C) tester; D., Data of F_1_s from 4133 Bt (D) tester; E., Data of F_1_s from SGK 9708 (E) tester; PS., Data of maternal lines

Significantly, associated loci were further scrutinized for determining the favorable alleles respective of their targeted traits on the basis of association results previously obtained. This phenotypic effect value was calculated through comparison between the average phenotypic value over genotypes with specified allele and that of all genotypes:

$$ ai=\sum xij/ ni-\sum Nk/ nk $$

However,

*ai*: phenotypic effect of the ith allele

x*ij*: phenotypic value over the jth accession with the *ith* allele

*ni*: number of accessions with the *ith* allele

*N*_k_: phenotypic value over all accessions

*n*_*k*_: number of accessions

If value for *a*_*i*_ came larger than zero then allele was considered with a positive effect, otherwise with negative effect.

## Additional files

Additional file 1: Estimates of the weighting coefficient (Eigen vector) associated with the principal components and different characters of Parents and F_1_s. (DOCX 19 kb)

Additional file 2: Summary of significant associations between markers and Phenotypic traits. (XLS 44 kb)

Additional file 3: Association of fiber quality and agronomic traits with microsatellites (XLS 56 kb)

Additional file 4: Association table displaying 46 microsatellites significantly (log10 > 3) associated with fiber quality and agronomic traits. (XLS 110 kb)

## Abbreviations

a_i_: Phenotypic effect
BN: Bolls per plant
Bt: *Bacillus thuringiensis*
BW: Boll weight
Dim: Dimension
DNA: Deoxyribonucleic acid
F_1_: First filial generation
FE: Fiber elongation
FL: Upper half mean length
FS: Fiber strength
FU: Fiber uniformity
FUI: Fiber uniformity index
GCA: General combining ability
HB: Heterobeltosis
HI: Heterosis index
hQTL: Heterosis related quantitative trait locus
K: Hypothetical number of subpopulations
K3: Competitive heterosis over check Rui za 816
K4: Competitive heterosis over check Eza mian 10hao (Tai D5)
L × T: Line into tester mating design
LD: Linkage disequilibrium
LnP(D): Log probability of data
LP: Lint percentage
Mb: Million base pairs
MIC: Fiber micronaire
MLM: Mixed linear model
MP: Mid-parent heterosis
PCA: Principle component analysis
PCR: Polymerase chain reaction
PH: Plant height
QTL: Quantitative trait locus
r: Correlation
*r*^*2*^: Coefficient of regression
SCA: Specific combining ability
