# Supplementary material for: Integration of conventional and advanced molecular tools to track footprints of heterosis in cotton
Source: BMC Genomics. 2018 Oct 29;19:776. doi: 10.1186/s12864-018-5129-4 (PMC6206862; doi:10.1186/s12864-018-5129-4)
Supplement: Supplementary file 1 — Estimates of the weighting coefficient (Eigen vector) associated with the principal components and different characters of Parents and F1s. (DOCX 19 kb) [file 12864_2018_5129_MOESM1_ESM.docx]

**Squared Cosines of Variables**

| **Traits** | **Eigen vectors** | | | | | | | | | | |
| --- | --- | --- | --- | --- | --- | --- | --- | --- | --- | --- | --- |
|  | **Dim-1** | **Dim -2** | **Dim -3** | **Dim -4** | **Dim -5** | **Dim -6** | **Dim -7** | **Dim -8** | **Dim -9** | **Dim-10** |  |
| **PH** | 0.0081 | **0.8018*** | 0.0024 | 0.0028 | 0.0024 | 0.0143 | 0.1505 | 0.0105 | 0.0070 | 0.0000 |  |
| **BW** | 0.0202 | **0.7773*** | 0.0099 | 0.0346 | 0.0007 | 0.0202 | 0.0707 | 0.0644 | 0.0021 | 0.0000 |  |
| **LP** | **0.3752*** | 0.0219 | 0.3068 | 0.0589 | 0.0085 | 0.1546 | 0.0459 | 0.0236 | 0.0046 | 0.0000 |  |
| **BN** | **0.3515*** | 0.0003 | 0.2421 | 0.1478 | 0.1545 | 0.0806 | 0.0105 | 0.0014 | 0.0114 | 0.0000 |  |
| **FL** | **0.6376*** | 0.0548 | 0.0623 | 0.0506 | 0.0437 | 0.0059 | 0.0057 | 0.0810 | 0.0581 | 0.0002 |  |
| **FS** | **0.7562*** | 0.0287 | 0.0611 | 0.0047 | 0.0082 | 0.0263 | 0.0035 | 0.0147 | 0.0945 | 0.0020 |  |
| **MIC** | **0.5197*** | 0.0379 | 0.0909 | 0.0826 | 0.1104 | 0.1191 | 0.0047 | 0.0300 | 0.0042 | 0.0006 |  |
| **FU** | 0.1923 | 0.0427 | **0.4879*** | 0.1936 | 0.0358 | 0.0022 | 0.0104 | 0.0018 | 0.0324 | 0.0009 |  |
| **FE** | 0.2176 | 0.0394 | **0.4049*** | 0.0409 | 0.2171 | 0.0004 | 0.0380 | 0.0404 | 0.0013 | 0.0000 |  |
| **FUI** | **0.8921*** | 0.0006 | 0.0641 | 0.0001 | 0.0283 | 0.0006 | 0.0029 | 0.0025 | 0.0011 | 0.0076 |  |
| **Eigenvalue** | 3.9706 | 1.8054 | 1.7323 | 0.6165 | 0.6095 | 0.4242 | 0.3431 | 0.2705 | 0.2166 | 0.0114 |  |
| **Total variance** | 39.7059 | 18.0540 | 17.3232 | 6.1652 | 6.0946 | 4.2415 | 3.4306 | 2.7047 | 2.1663 | 0.1140 |  |
| **% Cumulative** | 39.7059 | 57.7599 | 75.0831 | 81.2483 | 87.3428 | 91.5844 | 95.0149 | 97.7196 | 99.8860 | 100.0000 |  |
| Note: Values in bold and with ‘*’ correspond for each variable to the factor for which the squared cosine is the largest. | | | | | | | | | | | |

**Additional file 1:** Estimates of the weighting coefficient (Eigen vector) associated with the principal components and different characters of Parents and F_1_s.
